# Hepatitis B virus (HBV) serological patterns among the HBsAg negative hospital attendees screened for immunization

**DOI:** 10.1038/s41598-022-11535-8

**Published:** 2022-05-06

**Authors:** Hussein Mukasa Kafeero, Dorothy Ndagire, Ponsiano Ocama, Charles Drago Kato, Eddie Wampande, Abdul Walusansa, Henry Kajumbula, David Kateete, Hakim Sendagire

**Affiliations:** 1 Department of Medical Microbiology, College of Health Sciences, Makerere University, P. O Box 7062, Kampala, Uganda; 2 Department of Medical Microbiology, Habib Medical School, Faculty of Health Sciences, Islamic University in Uganda, P. O Box 7689, Kampala, Uganda; 3 Department of Plant Sciences, Microbiology and Biotechnology, College of Natural Sciences, Makerere University, P. O Box 7062, Kampala, Uganda; 4 Department of Medicine, College of Health Sciences, Makerere University, P. O Box 7062, Kampala, Uganda; 5 Department of Molecular Biology and Immunology, College of Health Sciences, Makerere University, P. O Box 7062, Kampala, Uganda; 6 Department of Biomolecular Resources and Biolab Sciences, College of Veterinary Medicine, Animal Resources and Biosecurity, Makerere University, P. O Box 7062, Kampala, Uganda

**Keywords:** Biomarkers, Diseases, Risk factors

## Abstract

The Hepatitis B virus (HBV) is a highly infectious virus and is endemic in Uganda. It is one of the major etiological agents for liver diseases including liver cancer. In this work, we evaluated the prevalence of the HBV serological markers and the associated socio-demographic factors among hepatitis B surface antigen (HBsAg) seronegative persons screened during routine immunization against the virus in eastern Uganda. Data on the socio-demographic characteristics were collected using a structured questionnaire, while that on the serological markers were obtained from serum samples and evaluated by using the 5-panel HBV One Step Hepatitis B Virus Combo Test Device (Fastep^R^, HBV-P43M). The following markers were evaluated by the panel: HBsAg, HBsAb, HBcAb, and HBeAb. Data were analyzed using SPSS (version 26), and multinomial logistic regression was used to elicit the adjusted odds ratio. All the analysis were performed at a 95% confidence limit, and a P value ≤ 0.05 was considered significant. The 424 participants included in this study were mainly female (62.3%), married (55.4%) and aged 30 years and above (54.2%). The seropositivity of the HBsAb, HBeAb, HBcAb marker prevalence rates was 48(11.3%), 73(17.2%) and 45(10.6%) respectively. The majority of the participants (327, 77.1%) did not present with any marker. Married paricipants were significantly associated with reduced HBsAb seropositvity rate, whereas young people aged 18–29 years were associated the with increased odds of HBsAb seropositivity (p < 0.05). Male participants were significantly associated with the HBeAb and HBcAb seropositivity (p < 0.05). Similarly, contact with an HBV infected person was significantly associated with HBeAb and HBcAb seropositivity (p < 0.05). Further still, blood transfusion was significantly associated with the increased risk of HBcAb seropositivity (P < 0.05). This study has revealed a prevalence of HBV serological markers among the HBsAg seronegative persons in this community and an increased risk of transmission of the virus in the community. Our findings have key consequences pertaining the interventions that are pertinent in the control and prevention of the spread of the virus among apparently health persons.

## Introduction

Hepatitis B virus (HBV) is the causative agent for liver inflammatory diseases, which, if not diagnosed in a timely manner and subsequently managed, are likely to progress to chronic liver diseases, liver fibrosis, liver cirrhosis, and liver cancer^1^. The virus has been implicated as one the most common oncogenic virus in humans^2^. It is a highly transmissible virus and is 50 to 100 times more infectious than Human Immune deficiency Virus (HIV). In addition, it has extreme resilience, allowing it to survive for several days on dry surfaces. This complicates its epidemiology and explains the increased chances of intra-familial horizontal transmissions^3^. Despite the presence of a safe and highly efficacious vaccine, HBV infection is still one of the major global health problems^4^. The Uganda Population-Based Impact Assessment (UPHIA) 2016–2017 survey reported a drastic decrease in the prevalence of HBV in Uganda^5^. According to this survey, the national prevalence of HBV dropped from 10% in the general population in 2015^6^ to 4.3% in 2016 and 4.1% in 2017, with east-central posting a prevalence of 2.1%. However, HBV is a chronic infection and these data are suggestive of either massive death of the chronically infected persons or a higher level of sero-conversion to HBsAb between the sampling intervals. Nevertheless, the rapid sero-conversion indicated by a high prevalence of the HBsAb with normal levels of the correlates of liver damage over a short period of time seems to be unrealistic. Similarly, the drastic decrease in the risk of infection because of improved immunity or public health awareness appears idealistic. The relative importance of the socio-demographic factors to HBV infection varies from population to population^7^, and their contribution to community spread of HBV have been previously reported with concordance in some studies and contradictions in other studies^8–12^. Understanding these sociodemographic factors related to infection and their relationship with markers of HBV exposure will provide plausible answers to the recent drastic decline in HBV in Uganda. To understand the sero-prevalence of hepatitis B virus, screening of a large number of people is needed. Serological markers for detection of HBV are diverse^13^ and include hepatitis B surface antibody (HBsAb), hepatitis B pre-core antibody (HBeAb), hepatitis B pre-core antigen (HBeAg), hepatitis B core antibody (HBcAb) and hepatitis B surface antigen (HBsAg). However, in resource limited settings, screening for hepatitis B virus infection is limited to only the HBsAg using the rapid diagnostic test. Unfortunately, the use of a single marker of exposure is associated with vast irregularities in the diagnosis of HBV. This inconclusive diagnosis is likely to mislead clinicians in their decisions when managing the HBV-infected persons as well as the decision to discard donated blood for transfusion by local and regional blood banks. HBV exposure markers have high sensitivity but low specificity, justifying the need to investigate them not in isolation for comprehensive case management and explicit assessment of blood for transfusion. The Anti-HBc is characteristic of a hidden HBV carrier state and/ or resolved disease^14^. Anti-HBs antibodies are associated with acquired immunity either due to previous exposure and natural response to the virus or due to vaccination^15^, whereas the Anti-HBe antibody is a marker of the minimally infective phase and disease remission or recovery from the infection^16^. Thus, as eluded from above, the relative significance of different sociodemographic risk factors for infection, the comparative expression of markers of liver damage and the relative prevalence of different markers of exposure to HBV at the community level can provide constructive clues on the trend of HBV prevalence and infectivity in a population. Consequently, we sought to understand the current state of the risk factors for HBV infection and the prevalence of the markers of immunity against HBV among HBsAg seronegative individuals to justify the drastic reduction in the burden of HBV in Uganda.

## Materials and methods

### Study area and period

A hospital-based study was conducted between January to September 2020 at Kibuku Health Center IV in eastern Uganda. This study site was chosen purposively because it was a pilot site for HBV vaccination in the eastern region during the study period. Kibuku District has a population of 250,600 with an area of 489.1 km^2^ and a population density of 512.4/km^2^^17^ at an elevation of 1100 m above sea level. Participants recruited in the study were from Kibuku District and the neighboring catchment areas.

### Study design and populations

We conducted a cross-sectional retrospective study with quantitative methods of data collection and analysis. Adult participants (age ≥ 18) were recruited from among outpatients coming to Kibuku Health Center IV for HBV screening before immunization during massive screening and vaccination against HBV as part of the government program to reduce HBV infection. These came from Kibuku District and the neighboring districts of Butebo, Budaka, Butaleja, Namutumba and Pallisa. Sampling was done from January 2020 to September 2020.

### Sample size determination and sampling procedure

The sample size (n) was estimated by using the formula described by Cochran^18^. A prevalence of HBV of 50% for no previously reported prevalence was used. Additionally, a standard normal deviation corresponding to the critical region of 1.96 at 5% precision was used. After correcting for the 10% loss due to unclear sample, a sample size of 394 was found to be sufficient. However, to raise the statistical power, an overall sample size of 424 participants was used. Purposive sampling was performed and any HBeAg sero-negative hospital attendee after screening was eligible for inclusion in the study.

### Eligibility criteria

Inclusion was based on HBsAg sero-negativity, having attained 18 years and above at the time of sampling as well as being a resident of Kibuku district and the sounding areas. Those who were below 18 years at the time of sampling were excluded from the study.

### Data collection and blood sampling

Demographic characteristics and predictors of HBV infection were collected by using a close-ended questionnaire administered by a nurse. In addition to the demographic characteristics of age, sex and marital status, the participants were asked whether they had ever transfused blood, consumed alcohol or had lived with HBV-infected persons before. The questionnaire was administered by a nurse or research assistant on site. For laboratory investigations, 4 mL of blood was drawn by vein puncture into anticoagulant vacutainers from which serum was obtained. The serum was kept in sterile viols and stored at −20 °C until further use.

### Serological testing

**Figure 1:**
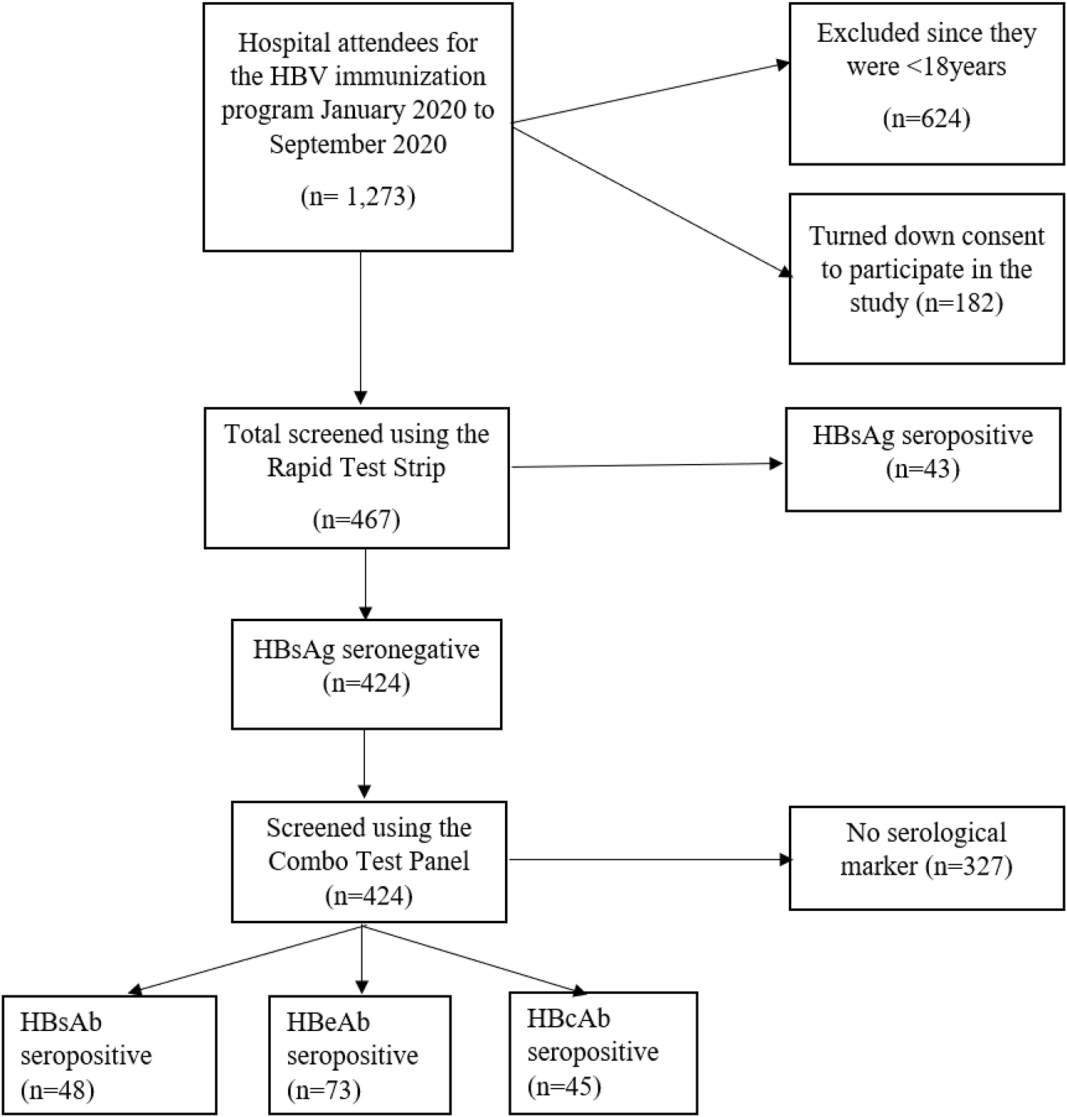
Screening algorithm over a period of 9 months; *HBV* hepatitis B virus, *HBsAg* hepatitis B surface antigen, *HBsAb* hepatitis B surface antibody, *HBeAb*  hepatitis B pre-core antibody, *HBcAb*  hepatitis B core antibody.

Screening for HBsAg serostatus was performed using the HBsAg Rapid Test Strip (Healgen Scientific Limited Liability Company, Houston, TX77047-USA), which uses a lateral flow chromatographic immunoassay for the qualitative detection of hepatitis B surface antigen (HBsAg) in human serum or plasma based on the double antibody-sandwich technique. For confirmation, the 5-panel HBV One Step Hepatitis B Virus Combo Test Device (Fastep^R^, HBV-P43 M) was used to confirm the HBsAg serostatus on all serum samples following the manufacturer’s instructions. In addition, the test was used for qualitative detection of other hepatitis B virus exposure markers, including HBsAb, HBeAg, HBeAb and HBcAb, in serum or plasma (Fig. 1).

### Data quality assurance

To ensure that the questionnaire was understandable, we pretested 5% of the questionnaires on the hospital attendees at Nsangi Health Center III and Kyengera Health Center III before the actual data collection at Kibuku Health Center IV. The collected data were checked daily for consistency and accuracy. Training was given to data collectors about data collection techniques. Finally, the positive result by the HBsAg Rapid Test Strip (Healgen Scientific Limited Liability Company, Houston, TX77047-USA) was rechecked by the 5-panel HBV One Step Hepatitis B Virus Combo Test Device (Fastep^R^, HBV-P43 M).

### Data presentation and statistical analysis

Categorical data are presented as frequencies and percentages. Univariate analysis was used to determine the crude odds ratio (COR), whereas multinomial logistic regression analysis was used to determine the adjusted odds ratio (AOR). All analyses were performed at the 95% level of significance, and p < 0.05 was considered to be statistically significant. Data were analyzed using SPSS version 26.

### Ethics declarations

The study protocol was approved by the Research and Ethics Committee of the School of Biomedical Sciences (SBS), College of Health Sciences (CHS), Makerere University; reference number SBS-REC-708 and the Uganda National Council for Science and Technology (UNCST); reference number HS575ES. The study was done following the guidelines from the Helsinki Declaration and all the participants provided written informed consent to participate in this study.

### Consent to participate

All participants provided written informed consent to participate in this study.

### Consent to publish

All participant’s identifiable data has been concealed by this article. Similarly, privacy rights for all participants have been observed and therefore the consent for publication was not applicable.

## Results

### Demographic characteristics and risk factors for infection with hepatitis B virus

As presented in Table 1, we had more female (62.3%) and married (55.4%) participants aged 30 years and above (54.2%).

**Table 1:** Summary of the sociodemographic characteristics of the study participants.

Variable	Categories	N	Marginal percentage (%)
Sex	Female	264	62.3
	Male	160	37.7
Married	No	189	44.6
	Yes	235	55.4
Age	≥ 30 years	230	54.2
	18–29 years	194	45.8
Blood transfusion	No	352	83.0
	Yes	72	17.0
Contact with infected person	No	314	74.1
	Yes	110	25.9
Alcohol use	No	311	73.3
	Yes	113	26.7

A total of 1273 reported for screening for HBsAg as a prerequisite for immunization against HBV over a period of 9 months at Kibuku Health center IV, of which 624 were below the age of consent and 182 turned down subsequent recruitment in the study. Forty-three participants were HBsAg seropositive representing a prevalence of 9.2% and 424 were HBsAg seronegative. There was 100% concordance between the two screening tests (Fig. 1).

**Figure 2:**
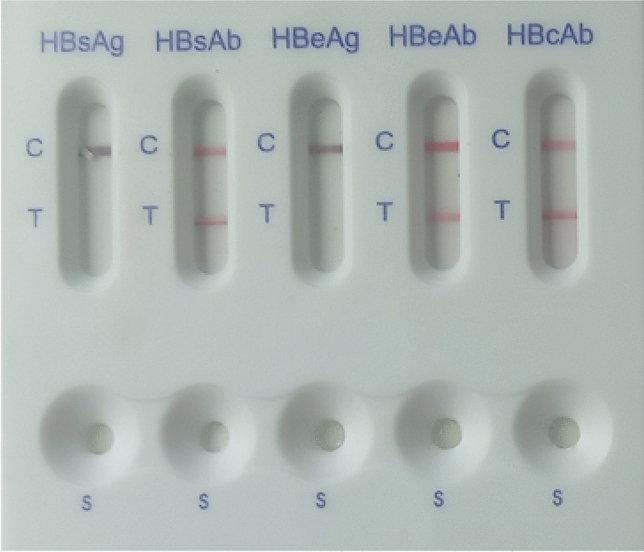
Representative results of the one step hepatitis B virus combo test cassette for the HBV serological markers among the HBsAg negative participants.

**Figure 3:**
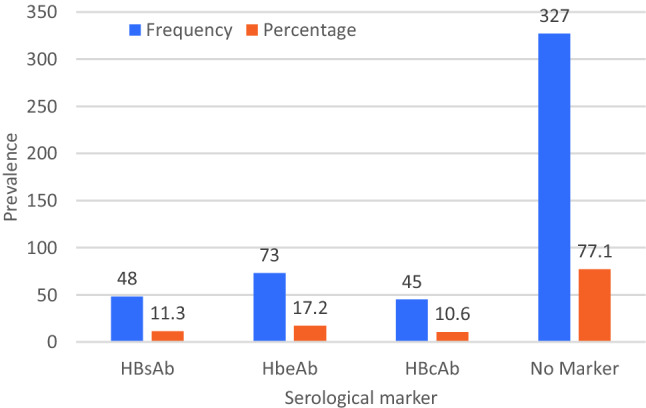
Serological profile of HBsAg negative outpatient study participants attending Kibuku Health Center IV.

The HBsAb, HBeAb, HBcAb seropositivity was detected among 48(11.3%), 73(17.2%) and 45(10.6%) participants respectively. In contrast, 327 (77.1%) of the participants did not present with any marker of past exposure to HBV as an infcetion or as a vaccine. (Figs. 2 and 3).

Interestingly, 3(0.71%), 26(6.1%) and 3(0.71%) were simultaneously positive for the two markers of HBsAb/HBeAb, HbeAb/HBcAb and HBsAb/HBcAb respectively. In addition, 12(2.8%) were concomitantly positive for the three markers of HBsAb/HBeAb/HBcAb (Table 2).

**Table 2:** Outline of the HBV serological profile of HBsAg seronegative participants screened from Kibuku Health center IV.

HBsAb	HBeAb	HBcAb	Number (%)
Negative	Negative	Negative	327 (77.1)
Positive	Negative	Negative	30 (7.1)
Positive	Positive	Negative	03 (0.71)
Negative	Negative	Positive	04 (0.94)
Negative	Positive	Positive	26 (6.1)
Positive	Negative	Positive	03 (0.71)
Negative	Positive	Negative	32 (7.6)
Positive	Positive	Positive	12 (2.8)

When we analysed the socio-demographic factors associated with the HBsAb serostatus by both univaraiate and multivariate logistic regression analysis, being married was significantly associated with reduced likelihood of being HBsAb seropositve (AOR = 0.255, 95%CI = [0.08 to 0.80], p = 0.021). In contrast, young people aged 18–29 years were almost 4 times likely to be HBsAb seropositive compared to those 30 years and above (AOR = 3.75, 95%CI = [1.61 to 8.739], p = 0.002) (Table 3).

**Table 3:** Socio-demographic factors associated with HBsAb serostatus among HBsAg seronegative hospital attendees screened for HBV before immunization.

Variable	Categories	HBsAb status		COR [95% CI]	AOR [95% CI]	P value
		Pos	Neg			
Sex	Male	3	45	1	1	
	Female	45	219	3.082 [0.92 to 10.35]	3.532 [0.798 to 15.64]	0.097
Marital status	Unmarried	34	155	1	1	
	Married	14	221	0.289 [0.15 to 0.56]	0.255 [0.08 to 0.80]	0.021*
Age (years)	≥ 30	10	220	1	1	
	18–29	38	156	5.359 [2.59 to 11.08]	3.751 [1.61 to 8.739]	0.002*
Blood transfusion	No	39	313	1	1	
	Yes	9	63	1.147 [0.529 to 2.49]	2.807 [0.698 to 11.288]	0.146
Contact with HBV infected person	No	34	280	1	1	
	Yes	14	96	1.201 [0.618 to 2.33]	1.222 [0.483 to 3.095]	0.672
Alcohol use	No	38	273	1	1	
	Yes	10	103	0.697 [0.335 to 1.45]	0.617 [0.139 to 2.741]	0.526

Concerning the analysis of the sociodemographic characteristics with HBeAb serostatus (Table 4), being male was significantly associated with HBeAb seropositivity (AOR = 5.773, 95%CI = [1.97 to 16.895], p = 0.001). Hence, male participants in our study were almost 6 times more likely to be HBeAb seropositive compared to the female participants. Similarly, contact with an HBV-infected person was significantly associated with HBeAb seropositive (AOR = 5.39, 95%CI = [2.421 to 12.00], p = 0.000).

**Table 4:** Socio-demographic factors associated with HBeAb serostatus among HBsAg seronegative hospital attendees screened for HBV before immunization.

Variable	Categories	HBeAb status		COR [95% CI]	AOR [95% CI]	P value
		Pos	Neg			
Sex	Female	32	232	1	1	
	Male	41	119	2.498 [1.496 to 4.17]	5.773 [1.97 to 16.895]	0.001*
Marital status	Unmarried	27	162	1	1	
	Married	46	189	1.46 [0.869 to 2.455]	1.207 [0.421 to 3.461]	0.726
Age (years)	≥ 30	47	183	1	1	
	18–29	26	168	0.603 [0.36 to 1.016]	0.730 [0.37 to 1.40]	0.355
Blood transfusion	No	62	290	1	1	
	Yes	15	57	1.23 [0.65 to 2.31]	1.14 [0.33 to 3.94]	0.836
Contact with HBV infected person	No	26	267	1	1	
	Yes	47	84	1.758 [1.027 to 3.01]	5.39 [2.421 to 12.00]	0.000*
Alcohol use	No	56	255	1	1	
	Yes	17	96	0.806 [0.446 to 1.46]	0.427 [0.153 to 1.187]	0.103

Pertaining the sociodemographic factors and HBcAb serostatus, being male was significantly associated with higher odds of HBcAb seropositivity (AOR = 5.8, 95%CI = [2.00 to 16.9], p = 0.001) than being female. Consequently, males were almost 6 times more likely to be HBcAb seropositive compared to their female counterparts. In addition, contact with HBV-infected persons was significantly associated with HBcAb seropositivity (AOR = 5.386, 95%CI = [2.42 to 11.989], p = 0.000) (Table 5).

**Table 5:** Socio-demographic factors associated with HBcAb serostatus among HBsAg seronegative hospital attendees screened for HBV before immunization.

Variable	Categories	HBcAb status		COR [95% CI]	AOR [95%CI]	P value
		Pos	Neg			
Sex	Female	13	251	1	1	
	Male	32	128	4.83 [2.448 to 9.517]	5.8 [2.00 to 16.90]	0.001*
Marital status	Unmarried	11	178	1	1	
	Married	34	201	2.737 [1.35 to 5.563]	1.207 [0.421 to 3.461]	0.762
Age (years)	≥ 30	28	202	1	1	
	18–29	17	177	0.69 [0.367 to 1.308]	0.727 [0.370 to 1.429]	0.36
Blood transfusion	No	37	315	1	1	
	Yes	8	64	1.064 [0.47 to 2.39]	3.907 [1.141 to 13.373]	0.030*
Contact with HBV infected person	No	14	283	1	1	
	Yes	31	96	6.53 [3.33 to 12.79]	5.386 [2.42 to 11.989]	0.000*
Alcohol use	No	34	277	1	1	
	Yes	11	102	0.879 [0.429 to 1.79]	0.301 [0.079 to 1.143]	0.078

Similarly, history of blood transfussion was significantly associated with increased odds of HBcAb seropositivity (AOR = 3.907, 95% CI = [1.141 to 13.373], p = 0.030). Thus, people with previous exposure to blood transfusion were close to 4 times more likely to be HBcAb seropositve when comaped to those who were blood transfusion naïve.

## Discussion

This is one of the first studies aimed at evaluating the serological markers of HBV infection and the role of the socio-demographic factors in influencing the prevalence of HBV serological markers in HBsAg seronegative persons screened for hepatitis B virus prior to immunization in a remote setting. Overall, 9.2% of the study participants were HBsAg seropositive. This prevalence is in fair conformity with the prevalence of 10% reported in 2015^6^ but much higher than the prevalence of 4.3% reported in 2016 and 4.1% in 2017^5^. However, the rapid decrease in the HBsAg seropositivity within an interval of three years appears more idealistic than realistic. None the less, our study was a hospital-based study and could have overestimated the prevalence of HBsAg. None of the HBsAg seronegative participant was HBeAg seropositive. The HBeAg is a marker of HBV active replication and viral transmission^19^ and it’s prevalence among the HBsAg seronegative persons would be elusive. In our study, we found that 11.3% of the HBsAg seronegative participants were HBsAb seropositive, which is an expression of immunity due to vaccination and/or naturally following exposure to the virus. Similarly, other markers of disease resolution, including the prevalence of HBcAb and HBeAb were reported to be 10.6% and 17.2%, respectively. Furthermore, simultaneous prevalence of two or more markers among our study participants were reported. Accordingly, 0.71%, 6.1%, 0.71% and 2.8% were concomitantly positive for HBsAb/HBeAb, HbeAb/HBcAb, HBsAb/HBcAb and HBsAb/HBeAb/HBcAb respectively. In contrast, most of our participants (77.1%) did not have any marker of HBV exposure and were susceptible to HBV infection.

The Anti-HBs antibodies prevalence rate reported in our study is lower than the prevalence of 22.2% reported by Mbaawuaga et al.^20^ among health persons in Nigeria and that of 28% reported by Goldsmith et al.^21^ among hospital personnel in Cairo, Egypt. These differences can be accounted for by various reasons: first, the differences in the vaccination coverage^22^, although our study did not determine the anti-HBs antibody titers to ascertain immunized participants; second, the differences in the infecting genotypes since particular genotypes are associated with better resolution of the infection than others^23–25^; and finally, the differences in host immunity^26,27^. Our results therefore suggest that there is an unmet need for vaccination in this community. Therefore, there is an urgent need to scale up vaccination in this community in particular and Uganda in general. In addition, genotyping of HBV in Uganda is not routine, so information on the infecting genotypes in Uganda is scant, yet it is important in guiding the implementation of the HBV control strategies^25^.

Furthermore, the anti-HBc prevalence rate reported in this study among the HBsAg seronegative is much higher than those reported from published studies among HBsAg seronegative blood donors in the developed countries of 2.3% in Saud Arabia^28^, 1.4% in German^29^, 2.1% in Iran^30^ but lower than the 15% reported by Zervou et al.^31^ in Greece and 13.5% reported by Hennig et al.^32^ in Korea. The HBcAb may be occult hepatitis B infection. In addition, the Anti-HBc seropositivity among the HBsAg seronegative has been implicated on mutations in the pre-core and core regions of the viral genome^33^. It is also conceivable that anti-HBc positive/HBsAg negative status is associated with the transmission of the virus through blood transfusion because persons with occult HBV are still infective^28,29^. Unfortunately, in anti-HBc positive individuals seronegative for HBsAg, the levels of HBV DNA are very low and require highly sensitive molecular methods to detect such low viremia, which may not be available in resource-constrained settings^34^. Furthermore, available evidence has implicated the anti-HBc positive/HBsAg negative condition in accelerating liver cirrhosis, end stage liver diseases and the risk of HBV reactivation among the immune compromised^34,35^.

The apparently high number of Anti-HBc seropositive individuals reported in our study and the studies from Greece and Korea compared to those reported from Saudi Arabia, German and Iran can be accounted for by the differences in the implementation of public health interventions like vaccination by the Governments in the respective countries. In addition, our study recruited hospital attendees coming for HBV screening before vaccination, whereas the aforementioned studies used blood donors. This difference in design could explain the differences in the results. In light of our findings, rigorous screening for anti-HBc positive/HBsAg negative in this community should be implemented especially among the risky groups, such as those immunocompromised with HBV-HIV co-infection or HBV-HIV-TB triple infection, to mitigate the likely progression to end-stage liver diseases. Similarly, blood should be screened for anti-HBc and then HBV-DNA before transfusion to avert the likelihood of transfusion of contaminated blood into potential recipients.

Furthermore, the anti-HBe antibody reported in our study was much higher than the prevalence of 0.4% among the HBsAg seronegative hospital attendees in Nigeria reported by Adetunji et al.^15^. The anti-HBe antibody is produced in response to HBeAg, and its manifestation in serum is a marker of minimally infective phase and disease remission or recovery from infection^16,36^. Anti-HBe antibodies usually appear a few weeks to a few months after HBsAg loss. At this stage a low level of viraemia is detectable by using molecular methods like polymerase chain reaction (PCR)^37^. Whereas the seroconversion of HBeAg to HBeAb is associated with remission from infection, it is also associated with complex stages of the viral replicative cycle, such as integration of the viral genome into the host DNA. Moreover, the integration has been implicated in triggering the onset of hepatocellular carcinoma (HCC)^38^. Thus, our results on HBeAb + prevalence in this community have serious public health concerns and should not be taken lightly. Hence, the followings mitigations are paramount: first, the screening of the donated blood for HBeAb and later confirming the absence of the viral particles using PCR should be a prerequisite before transfusing the blood into recipients, and second, the monitoring of the patients with Anti-HBe antibodies for early detection and management of HCC should be a necessity. None the less, it is plausible that the concomitant prevalence of two or more seropositive positive markers generally presuppose immunity due to previous natural exposure to the virus and later recovery or a result of vaccination^19^.

Furthermore, our results also reported that majority of the participants (77.1%) did not have any marker of previous HBV exposure in form of vaccination or acquisition of infection and later recover and were hence prone to HBV infection. With this high percentage of people susceptible to HBV infection, the eradication of HBV in this population is a mystery as highlighted by the sustainable development goals. This prevalence was much higher than those reported in other countries where community vaccination against HBV is presumably high. For example, Adetunji et al.^15^ reported 57.7% health hospital attendees in Nigeria were immunization naïve whereas Ifeorah et al.^39^ reported that only 47.4% of pregnant women on antenatal care were immunization naïve. The discrepancy observed in the results of our study and those from studies elsewhere can be explained in terms of the differences in the implementation of the vaccination programs by the respective health systems. Therefore, our results suggest that the prevalence of this large number of people at high risk of HBV infection in this community is indicative of an unmet need for vaccination coverage.

The concomitant prevalence of HBsAb + /HBcAb + , HBsAb + /HBeAb, HBcAb + /HBeAb + and HBsAB + /HBcAb + /HBeAb + reported in our study is an indicator of previous natural exposure to the virus^15,19,20^. The generally low prevalence of duo or trio positive markers is in conformity with the high prevalence of many individuals in this community who are unexposed to HBV contrary to the findings elsewhere^15,19,20^. This finding should be of public health concern because many people are prone to infection with HBV in this community and hence scalling up immunization should be priotilized.

Regarding the relative prevalence of the markers by age group, this study established that the prevalence of HBsAb was significantly higher among the young people (p < 0.05). The association between age and HBV infection has been earlier reported^40,41^. The prevalence of HbsAb among the young people could be partly associated with child hood immunization against the virus in this cohort. Moreover, the inclusion of the HBV vaccine as part of the expanded program on immunization (EPI) in Uganda was introduced in 2002^42^, and some of the participants born thereafter were included in our study.

We also found a significant association between the HBeAb and HBcAb markers and sex in this study (P < 0.05), with males having higher seroprevalence of both markers than females. These results are consistent with the findings by Mohammed et al.^19^ in Nigeria and Bwogi et al.^43^ in Uganda. This can be attributable to the indulgence of men in risky sexual behaviors compared to women, as earlier reported in the study by Omatola et al.^44^. Unfortunately, men have low clearance rate of HBsAg, which increases the risk of progression to end-stage disease such as liver cirrhosis and hepatocellular carcinoma^45^. Consequently, our results suggest that men should pioneer the campaign against HBV in Uganda because they are at a higher risk than their female counterparts.

For the other socio-demographic characteristics, interesting relationships between the HBsAb, HBeAb and HBcAb were observed. First, being married was significantly associated with reduced odds of HBsAb seropositivity (P < 0.05) suggesting an unmet need for HBsAg screening and subsequent vaccination among prospective couples prior to marriage. Moreover, the prevalence of HBsAb seropositivity was higher among the young participants who are less likely to be married. Second, blood transfusion was significantly associated with increased chances of HBcAb seropositivity (P < 0.05) in conformity with the report by Mohammed et al.^19^. Thus, despite the recommendation by the World Health Organization (WHO) to screen the donated blood for HBV before transfusion, there is an unmet need to effectively screen the blood, increasing the risk of transmission of the virus along with the transfused blood. Third, the history of house hold contact with an HBV positive person was 5 times associated with anti-HBe and anti-HBc seropositivity significantly higher than those without prior house hold contact (P < 0.05). The presence of an HBV carrier member in the family has been reported to increase the risk of HBV transmission by 11–57% in studies by Ragheb et al.^46^ in Egypt, Lobato et al.^47^ in Brazil and Sofian et al.^48^ in Iran. This is due to the highly contagious nature of the virus making it 100 times more contagious than HIV and can stay on the surfaces for 7 days^49^. Therefore, as illuminated by our study, surveillance of homesteads with the aim of establishing any possible HBV-infected persons should be part of the routine work of the village health team (VHT) members. Once identified, family members should be trained on the aspects of their management to reduce household and probably community spread of the virus.

Finally, it should be noted that alcohol use did not affect the prevalence of anti-HBs, anti-HBe and anti-HBc among the HBsAg seropositive individuals, contrary to earlier report by Mabry-Hernandez et al.^50^ which implicated alcohol as a key driver in the transmission of HBV. However, the alcohol abuse was self-reported, and probably the participants were not sincere with this habit, and this could have affected the accuracy of our results.

In conclusion, though Uganda is an HBV endemic country, the prevalence of markers of HBV infection has not been well documented. In our study, we have identified the serologic markers of HBV infection and analyzed them in tandem with the socio-demographic in a group of apparently health people screened prior to immunization in eastern Uganda. Our results suggest that a proportionately small number of people have had exposure to the virus and have resolved the infection whereas majority were naïve. Men and persons who have had a family member with HBV had more exposure. Our results have highlighted an empirical dilemma and an uphill task towards the management of HBV in Uganda. The task ranges from rigorous screening for markers of exposure in the general population, through thorough evaluation of blood for transfusion to massive vaccination of adults without any of the markers of exposure. In light of our findings, the drastic decrease in HBV prevalence reported by the Uganda Population Health Impact Assessment (UPHIA) over the last 5 years appears idealistic rather than realistic, although more studies are warrantied on a large scale for better country-wide representation.

Our study could not go without limitations:—first, the retrospective study design which is liable to recall bias, secondly, the self-reported alcohol intake may have led to unrealistic responses and finally, the hospital-based nature of the study could not give results that can be generalized to the general population.

## Data Availability

All data generated or analyzed during this study are included in this published article.

## References

[1] Matsuura K, Tanaka Y, Hige S, Yamada G, Murawaki Y, Komatsu M (2009). Distribution of hepatitis B virus genotypes among patients with chronic infection in Japan shifting toward an increase of genotype A. J. Clin. Microbiol..

[2] Nguyen VT, Law MG, Dore GJ (2008). An enormous hepatitis B virus-related liver disease burden projected in Vietnam by 2025. Liver Int..

[3] Weinbaum C, Williams I, Mast E, Wang S, Finelli L, Wasley A (2008). Recommendations for identification and public health management of persons with chronic hepatitis B virus infection. MMWR Recomm. Rep..

[4] Ganem D, Prince A (2004). Hepatitis B virus infection–natural history and clinical consequences. N. Engl. J. Med..

[5] UPHIA. *UPHIA. Uganda Population HIV Impact Assessment*. (2019).

[6] MOH. *Ministry of Health Uganda, 2015 Press Statement on the World Hepatitis Day by the Ministry of Health Ugandan Government*. Ps@health.go.ug. (2015).

[7] Frambo A, Atashili J, Fon P, Ndumbe P (2014). Prevalence of HBsAg and knowledge about hepatitis B in pregnancy in the Buea Health District, Cameroon: A cross-sectional study. BMC Res. Notes..

[8] Bani I, Mahfouz M, Maki E, Gaffar A, Elhassan I, Yassin A (2012). Prevalence and risk factors of hepatitis B virus among pregnant women in Jazan Region-Kingdom of Saudi Arabia. J. Biol. Agric. Heal..

[9] Kafeero HM, Ndagire D, Ocama P, Kudamba A, Walusansa A, Sendagire H (2021). Prevalence and predictors of hepatitis B virus ( HBV ) infection in east Africa : Evidence from a systematic review and meta-analysis of epidemiological studies published from 2005 to 2020. Arch. Public Heal..

[10] Eke A, Eke U, Okafor C, Ezebialu I, Ogbuagu C (2011). Prevalence, correlates and pattern of hepatitis B surface antigen in a low resource setting. Virol. J..

[11] Awole M, Gebre-Selassie S (2005). Seroprevalence of HBsAg and its risk factors amoung pregnant women in Jimma, Southwest Ethiopia. Ethiop. J. Heal. Dev..

[12] Lawal MA, Adeniyi OF, Akintan PE, Salako AO, Omotosho OS, Temiye EO (2020). Prevalence of and risk factors for hepatitis B and C viral co-infections in HIV infected children in Lagos, Nigeria. PLoS ONE.

[13] Mahoney FJ (1999). Update on diagnosis, management, and prevention of hepatitis B virus infection. Clin. Microbiol. Rev..

[14] Lok AS, Cl L, Wu PC (1988). Prevalence of isolated antibody to hepatitis B core antigen in area endemic for hepatitis B virus infection: Implications in hepatitis B vaccination programme. Hepatology.

[15] Adetunji SO, Donbraye E, Alawode- L, Akinniyi O (2018). Serological profile of HBV infection among apparently healthy hospital attendees. J. Immunoassay Immunochem..

[16] Mast E, Weinbaum C, Fiore A, Alter M, Bell B (2006). A comprehensive immunization strategy to eliminate transmission of hepatitis B virus infection in the United States: Recommendations of the advisory committee on immunization practices (ACIP) part II: Immunization of adults. MMWR Recomm. Rep..

[17] UBOS. *National Population and Housing Census 2014-Area Specific Profiles. The Repuloc of Uganda, Kampala [Internet]*. ubos.org/wp-content/uploads/publications/2014CensusProfiles. (2017).

[18] Cochran WG (1977). Sampling Techniques.

[19] Mohammed HI, Pennap GR, Oti VB, Adoga MP (2019). Markers of hepatitis B virus infection in a subset of young people in central Nigeria. Sci. Afr..

[20] Mbaawuaga E, Iroegba C, Ike A (2014). Hepatitis B virus serological patterns in Benue state, Nigeria. Open J. Med. Microbiol..

[21] Goldsmith R, Zakaria S, Zakaria M, Mabrouk M, Hanafy A, El-Kaliouby A (1989). Occupational exposure to hepatitis B virus in hospital personnel in Cairo, Egypt. Acta Trop..

[22] Yun-Fan L, Chia-Ming C (2009). Hepatitis B virus infection. Lancet.

[23] Wai C, Chu C, Hussain M (2002). HBV genotype B is associated with better response to interferon therapy in HBeAg (+) chronic hepatitis than genotype C. Hepatol. Hepatol..

[24] Erhardt A, Blondin D, Hauck K, Sagir A, Kohnle T, Heintges T (2005). Response to interferon alfa is hepatitis B virus genotype dependent: Genotype A is more sensitive to interferon than genotype D. Gut.

[25] Kafeero HM, Ndagire D, Ocama P, Kato CD, Wampande E, Kajumbula H (2022). Disproportionate distribution of HBV genotypes A and D and the recombinant genotype D/E in the high and low HBV endemic regions of Uganda: A wake-up call for regional specific HBV management. Int. J. Hepatol..

[26] Kafeero M, Sendagire H, Ocama P, Ndagire D (2019). Host and viral factors associated with hepatitis B clinical outcomes in chronic infection-review article. Int. J. Pure Med. Res..

[27] Kafeero HM, Ndagire D, Ocama P, Walusansa A, Sendagire H (2022). Tumor necrosis factor-α-863C/A and 1031 T/C single nucleotide polymorphic sites (SNPs) may be putative markers of HBV disease prognosis among Caucasoids: Evidence from a systematic review with meta-analysis. Gene Rep..

[28] Alshayea AI, Eid GE, El-Hazmi MM, Alhetheel AF (2016). Prevalence and characterization of occult hepatitis B infection among blood donors in central Saudi Arabia. Saudi Med. J..

[29] Kleinman S, Kuhns M, Todd D, Glynn S, McNamara A, DiMarco A (2003). Frequency of HBV DNA detection in US blood donors testing positive for the presence of anti-HBc: Implications for transfusion transmission and donor screening. Transfusion.

[30] Sofian M, Aghakhani A, Izadi N, Banifazl M, Kalantar E, Eslamifar A (2010). Lack of occult hepatitis B virus infection among blood donors with isolated hepatitis B core antibody living in an HBV low prevalence region of Iran. Int. J. Infect. Dis..

[31] Zervou E, Dalekos G, Boumba D, Tsianos E (2001). Value of anti-HBc screening of blood donors for prevention of HBV infection: Results of a 3-year prospective study in Northwestern Greece. Transfusion.

[32] Hennig H, Puchta I, Luhm J, Schlenke P, Goerg S, Kirchner H (2002). Frequency and load of hepatitis B virus DNA in first-time blood donors with antibodies to hepatitis B core antigen. Blood.

[33] Marusawa H, Uemoto S, Hijikata M, Ueda Y, Tanaka K, Shimotohno K (2000). Latent hepatitis B virus infection in healthy individuals with antibodies to hepatitis B core antigen. Hepatology.

[34] Raimondo G, Locarnini S, Pollicino T, Levrero M, Zoulim F, Lok AS (2019). Update of the statements on biology and clinical impact of occult hepatitis B virus infection. J. Hepatol..

[35] Chang JJ, Mohtashemi N, Bhattacharya D (2018). Significance and management of isolated hepatitis B core antibody (anti-HBc) in HIV and HCV: Strategies in the DAA era. Curr. HIV AIDS Rep..

[36] Kao J (2008). Diagnosis of hepatitis B virus infection through serological and virological markers. Expert Rev. Gastroenterol. Hepatol..

[37] Lieberman HM, LaBrecque DR, Kew MC, Hadziyannis SJ, Schafritz DA (1983). Detection of hepatitis B virus DNA directly in human serum by simplified molecular hybridization test: Comparison to HBeAg/anti-HBe status in HBsAg carriers. Hepatology.

[38] Shfritz DA, Lieberman HM (1984). The molecular biology of hepatitis B virus. Annu. Rev. Med..

[39] Ifeorah IM, Bakarey AS, Adewumi MO, Faleye TOC, Akere A, Omoruyi CE (2017). Patterns of serologic markers of hepatitis B virus infection and the risk of transmission among pregnant women in southwestern Nigeria. J. Immunoass. Immunochem..

[40] Ochola E, Ocama P, Orach CG, Nankinga ZK, Kalyango JN, McFarland W, Karamagi C (2013). High burden of hepatitis B infection in Northern Uganda : Results of a population-based survey. BMC Public Health.

[41] Stabinski L, Reynolds SJ, Ocama P, Laeyendecker O, Boaz I, Ndyanabo A (2011). High prevalence of liver fibrosis associated with HIV infection: A cross-sectional study in rural Rakai, Uganda. Antivir. Ther..

[42] WHO. *World Health Organization: Uganda Reported Immunization Coverage*. https://www.who.int/immunization_monitoring/en/%0Aglobalsummary/timeseries/%0Atscoveragebycountry.cfm?country=Uganda. Accessed 16 June 2007 (2007).

[43] Bwogi J, Braka F, Makumbi I, Mishra V, Bakamutumaho B, Nanyunja M, Opio A (2009). Hepatitis B infection is highly endemic in Uganda: Findings from a national serosurvey. Afr. Health Sci..

[44] Omatola, C.A., Onoja, B.A., Agama, J. Detection of hepatitis B surface antigen among febrile patients in Ankpa, Kogi State, Nigeria. *J. Trop. Med*. (2020). 10.1155/2020/5136785 PMC7036110 32095141

[45] Martinson F, Weigle K, Royce R (1998). Risk factors for horizontal transmission of hepatitis B in a rural district in Ghana. Am. J. Epidemiol..

[46] Ragheb M, Elkady A, Tanaka Y, Murakami S, Attia F, Hassan A (2012). Multiple intra-familial transmission patterns of hepatitis B virus genotype D in north-eastern Egypt. J. Med. Virol..

[47] Lobato C, Tavares-Neto J, Rios-Leite M, Trepo C, Vitvitski L, Parvaz P (2006). Intrafamilial prevalence of hepatitis B virus in Western Brazilian Amazon region: Epidemiologic and biomolecular study. J. Gastroenterol. Hepatol..

[48] Sofian M, Banifazl M, Ziai M, Aghakhani A, Farazi AA, Ramezani A (2016). Intra-familial transmission of hepatitis B virus infection in Arak, central Iran. Iran J Pathol..

[49] EMI (2016). Hepatitis B virus: epidemiology and transmission risks. EMI Guidel..

[50] Mabry-Hernandez I, Lewis P (2015). Screening for hepatitis B virus infection in nonpregnant adolescents and adults. Am. Fam. Phys..

